# First clinical application of image-guided intraoperative electron radiation therapy with real time intraoperative dose calculation in recurrent rectal cancer: technical procedure

**DOI:** 10.1186/s13014-023-02374-6

**Published:** 2023-11-10

**Authors:** Falk Roeder, Gerd Fastner, Christoph Fussl, Felix Sedlmayer, Markus Stana, Johannes Berchtold, Tarkan Jäger, Jaroslav Presl, Philipp Schredl, Klaus Emmanuel, Daniela Colleselli, Gabriel Kotolacsi, Philipp Scherer, Philipp Steininger, Christoph Gaisberger

**Affiliations:** 1 Department of Radiation Therapy and Radiation Oncology, Paracelsus Medical University, Müllner Hauptstrasse 48, 5020 Salzburg, Austria; 2 Institute of Research and Development of Advanced Radiation Technologies (radART), Paracelsus Medical University, Müllner Hauptstrasse 48, Salzburg, Austria; 3 Department of Visceral and Thoracic Surgery, Paracelsus Medical University, Müllner Hauptstrasse 48, Salzburg, Austria; 4 Department of Urology, Paracelsus Medical University, Müllner Hauptstrasse 48, Salzburg, Austria; 5 Department of Anesthesiology, Paracelsus Medical University, Müllner Hauptstrasse 48, Salzburg, Austria

**Keywords:** Recurrent rectal cancer, Intraoperative radiation therapy, Image-guided radiation therapy

## Abstract

Intraoperative radiation therapy (IORT) is a radiation technique applying a single fraction with a high dose during surgery. We report the first abdomino-pelvic application of an image-guided intraoperative electron radiation therapy with intraoperative real time dose calculation based on the individual intraoperative patient anatomy. A patient suffering from locoregionally recurrent rectal cancer after treatment with neoadjuvant re-chemoradiation was chosen for this approach. After surgical removal of the recurrence, an adequate IORT applicator was placed as usual. A novel mobile imaging device (ImagingRing, MedPhoton) was positioned around the patient covering the region to be treated with the IORT-applicator in place. It allowed the acquisition of three-dimensional intraoperative cone-beam computed tomography images suitable for dose calculation using an automated scaling (heuristic object and head scatter as well as hardening corrections) of Hounsfield units. After image acquisition confirmed the correct applicator position, the images were transferred to our treatment planning system for intraoperative dose calculation. Treatment could be accomplished using the calculated dose distribution. We herein describe the details of the procedure including necessary adjustments in the typically used IORT equipment and work flow. We further discuss the pros and cons of this new approach generally overcoming a decade long limitation of IORT procedures as well as future perspectives regarding IORT treatments.

## Introduction

Intraoperative electron radiation therapy (IOERT) is a radiation therapy technique applying a single fraction with a high dose via electrons during surgery [1, 2]. It is used mainly as a boosting technique combined with pre- or postoperative external beam radiation therapy (EBRT) in situations with a high risk of local failure but limited opportunities for further external dose escalation due to surrounding organs at risk with low radiation tolerance [1–4]. Classic examples include abdominopelvic malignancies such as locally advanced or locally recurrent pancreatic cancer [5–7], colorectal cancer [8–10] or (retroperitoneal) soft-tissue sarcoma [11, 12]. Limiting adjacent organs at risk include mainly bowel, stomach, or kidneys, which is even more true if the tumor had recurred after prior irradiation. The main advantage of an IOERT boost is to overcome these dose limitations by simply moving the adjacent organs at risk out of the irradiation area during surgery.

Although IOERT has been used for more than 4 decades, the technique itself has little changed since its introduction. After resection, an applicator of adequate size and shape to cover the tumor bed or residual disease (if present) with an axial safety margin is placed intraoperatively by the surgeon together with the radiation oncologist [1, 2]. Critical organs at risk with low radiation tolerance (for example small bowel) are surgically moved outside the irradiation area [1, 2]. The central axis of the applicator is then properly aligned with a mobile or dedicated electron linear accelerator available in the operation room either by moving the patient or the accelerator [1, 2]. The tissue depth which has to be covered is approximated by probe measurements or intraabdominal ultrasound [1, 2]. An adequate electron energy is chosen to cover the measured tissue depth by the 90% isodose [1, 2] on the central axis. During irradiation, the patient is monitored via video. After irradiation, the applicator is removed and the surgical procedure is finished similarly to a non-IOERT intervention.

One of the main limitations of IOERT approaches so far was the inability to perform three-dimensional treatment planning and visualization of the dose distribution based on the individual patient anatomy [1] as it has been standard in EBRT for decades. Dose prescription mainly relied on tabulated values based on water phantom measurements for the different sizes and shapes of the available applicators with the different available electron energies [1–3]. Some advanced centers were capable of estimating the dose distribution in the individual patient based on preoperative images using an approximation of the applicators position during surgery (so called virtual planning) [13, 14], but no real time treatment planning during surgery based on the intraoperative anatomy was possible. Therefore, possible changes in dose distribution due to tissue inhomogeneities or surface irregularities could not be taken into account. Moreover, no confirmation or documentation of the correct applicator position was achievable beside the experience and judgement of the treating surgeon and radiation oncologist due to a lack of adequate intraoperative imaging.

A variety of theoretical and clinical attempts to overcome at least some of these limitations have been made over the past 2 decades [15]. Different imaging techniques like surface scanning by stereoscopic cameras, orthogonal X-rays, or ultrasound have been investigated within phantom studies or in vivo to at least confirm a correct applicator position or to allow some kind of a more sophisticated treatment planning [16–22], but all fell short in solving the main issue. More accurate approximations were reported by Garcia-Vazquez et al. [23, 24]: In a phantom study, they performed virtual planning on preoperative CTs as a gold standard and evaluated the technical usability of intraoperative images acquired by different kV- and MV-conebeam CT scanners for dose calculation by comparison. They concluded that two of the systems would be suitable [23]. In a clinical study, they evaluated six sarcoma patients who received IORT using a similar virtual planning strategy. Moreover, those patients were transported intraoperatively from the operating room to a non-dedicated CT scanner to acquire intraoperative images with the applicator in place. Those images were rigidly registered with the preoperative images to account for deviations of the actual and the simulated applicator position especially regarding possible air gaps. Dose calculations on the intraoperative and the preoperative scans were compared for three patients without major metal artifacts. They concluded that conventional assumptions of water-equivalent tissues or the use of preoperative scans only may lead to inaccurate IOERT dose distributions [24], thus strengthen the rationale for IOERT dose calculations based on intraoperative imaging.

In summary, all of the mentioned approaches were either not able to solve the problem of an accurate dose calculation for IOERT or needed transportation of the patient outside of the operation theatre, which would be a major drawback in the era of mobile or dedicated LINACS. Mobile CBCT-scanners seem to be an ideal addition to enable appropriate real-time intraoperative dose calculation of IOERT procedures. Recently, such a mobile CBCT scanner (ImagingRing, medPhoton) has been introduced into our IOERT suite and can be used for intraoperative image-guidance and real time dose calculation. We present the first clinical application of an image-guided abdomino-pelvic IOERT with real time dose calculation in a patient suffering from recurrent rectal cancer with an emphasis on the technical procedure and the clinical work-flow.

## Case

**Fig. 1:**
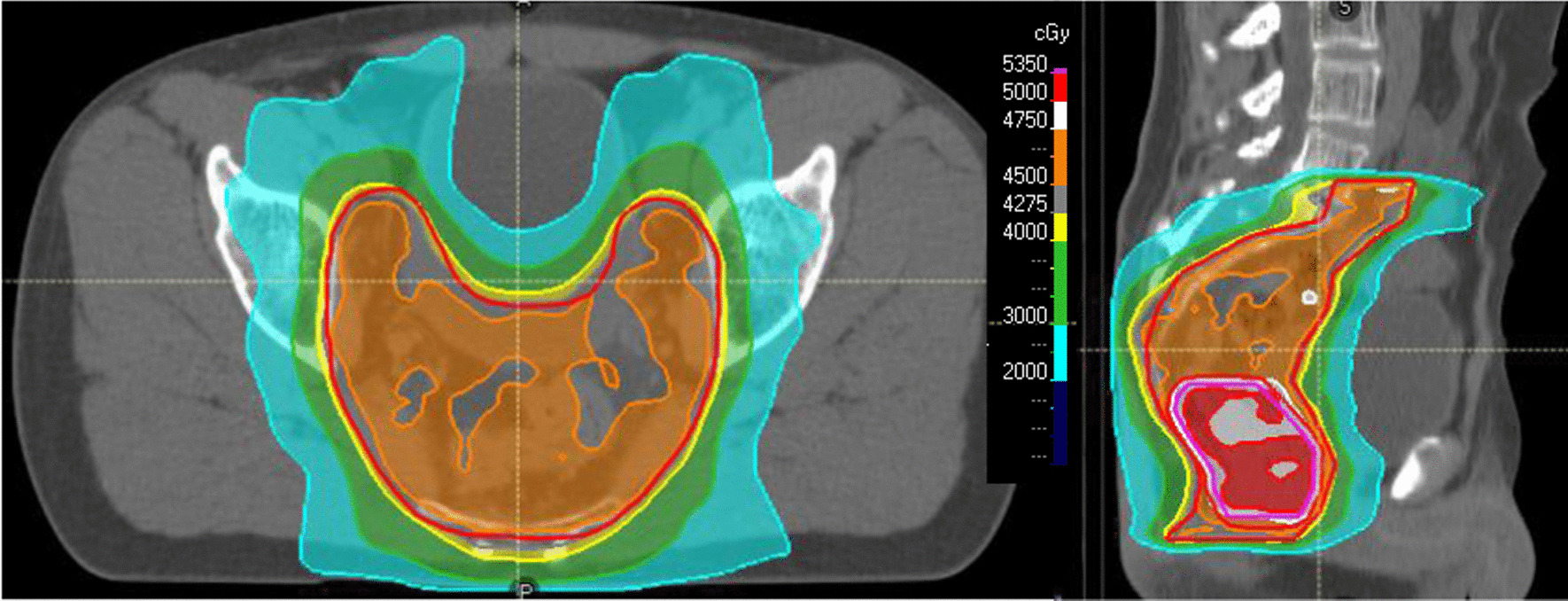
Dose distribution of initial neoadjuvant irradiation (prescription dose 45 Gy to pelvic nodal regions, 50 Gy simultaneous-integrated boost to primary tumor region), left: axial, right: sagittal

**Fig. 2:**
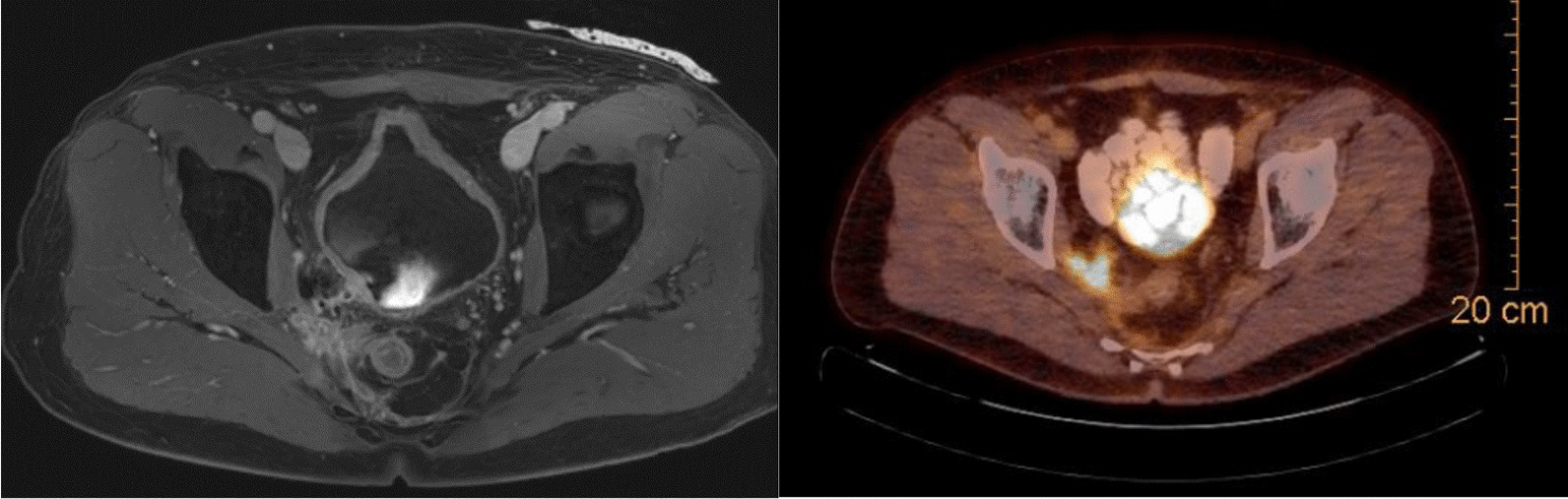
T1-weighted MRI (left) and FGD-PET CT with pelvic side wall recurrence prior to re-irradiation (right)

**Fig. 3:**
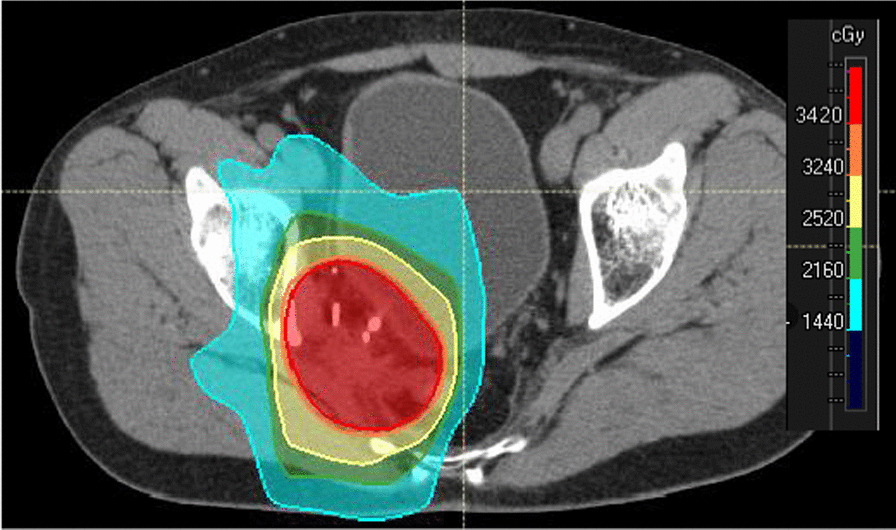
Dose distribution of neoadjuvant pelvic re-irradiation (left, prescription dose 36 Gy)

We report on a 49 year old male patient suffering from locally-recurrent rectal cancer. Initial diagnosis of locally advanced rectal cancer was made in 2019. Initial staging revealed microsatellite stable, well differentiated adenocarcinoma staged cT3bcN0cM0 by endoscopy, pelvic magnetic resonance imaging (MRI) and chest/abdominal computed tomography (CT). He received neoadjuvant radiation using image-guided volumetric-intensity modulated arc therapy (VMAT) with 45 Gy (single dose 1.8 Gy) to the pelvic nodal regions and 50 Gy (single dose 2 Gy) to the primary tumor at our center (Fig. 1). Radiation therapy was combined with capecitabine (825 mg/m^2^ twice daily on RT treatment days). Surgery was performed 6 weeks later by transanal minimally invasive total mesorectal excision at the referring center including protective ileostomy. Final pathology revealed an ypT3ypN0 stage with negative circumferential margin. The patient received adjuvant chemotherapy with capecitabine/oxaliplatin at the referring center, which was later reduced to capecitabine mono. He remained tumor-free during follow-up for 18 months and received re-anastomosis of the ileostomy 2 years after first diagnosis. Unfortunately, he developed total stool incontinence and was finally treated with a permanent colostomy. One month later, a local recurrence in the right pelvic side wall was diagnosed. Staging further revealed a small solitary pulmonary metastasis. The local recurrence was treated by laparoscopic resection at the referring center but final pathology revealed multifocal microscopic incomplete resection. The patient was scheduled for postoperative re-irradiation and again referred to our center. Restaging with MRI and 18-F-fluorodeoxyglucose positron emission tomography computed tomography (FDG-PET-CT) confirmed a small, still solitary pulmonary metastasis, but showed gross residual tumor in the right pelvic side wall (Fig. 2). After multidisciplinary discussion and individual counseling of the patient, we decided to offer total neoadjuvant therapy consisting of dose-reduced re-chemoradiation, consolidative chemotherapy, and attempted surgery with IOERT. Neoadjuvant chemoradiation consisted of image-guided VMAT to the recurrent tumor with small safety margins up to a dose of 36 Gy (single dose 1.8 Gy) with concurrent capecitabine (Fig. 3). Afterwards, the patient received 6 cycles of consolidation chemotherapy with capecitabine/oxaliplatin. The pulmonary metastasis was treated with ablative stereotactic body radiation therapy in 3 fractions of 15.4 Gy (prescribed to the surrounding 65% isodose). He was then scheduled for surgery including IOERT 6 weeks after the end of chemotherapy.

### Surgery and image-guided IOERT (technical procedure)

**Fig. 4:**
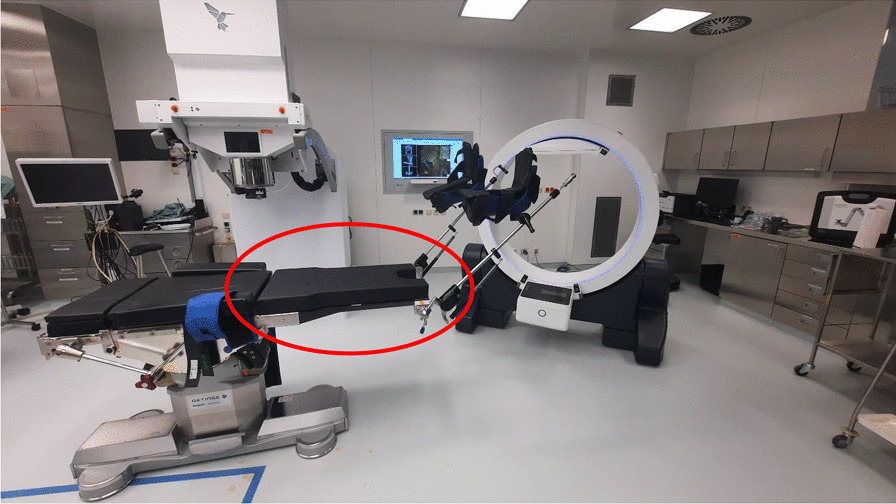
Carefully selected operating table setup with fully X-rays capable part between table Column and leg positioning device

**Fig. 5:**
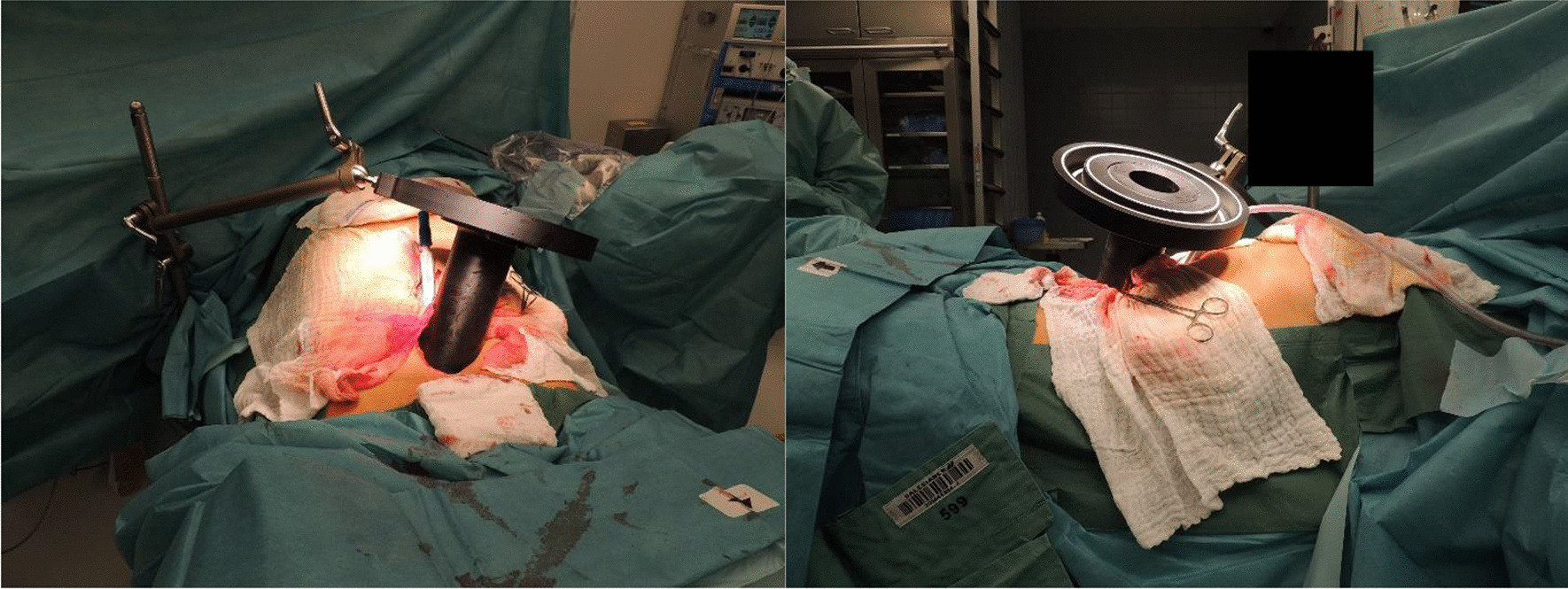
IOERT-Applicator placed in treatment position

**Fig. 6:**
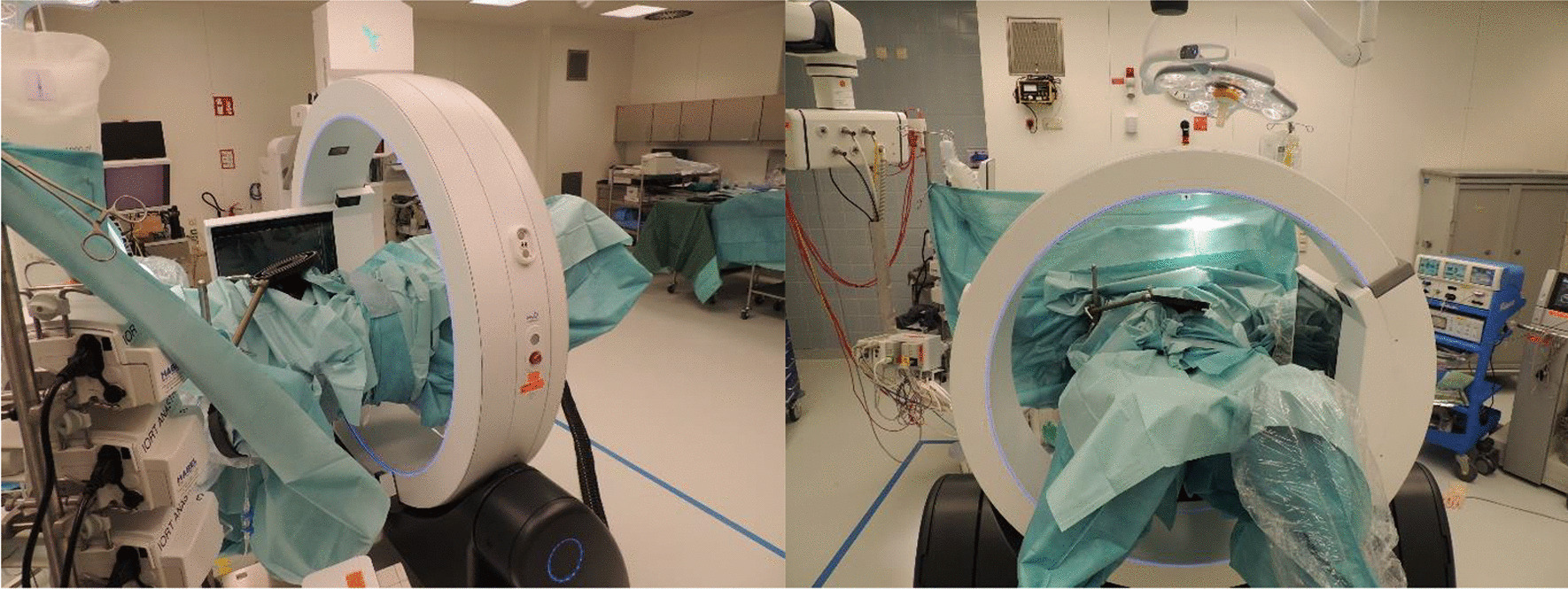
Patient wrapped in sterile covers with mobile ImagingRing in image acquisition position

**Fig. 7:**
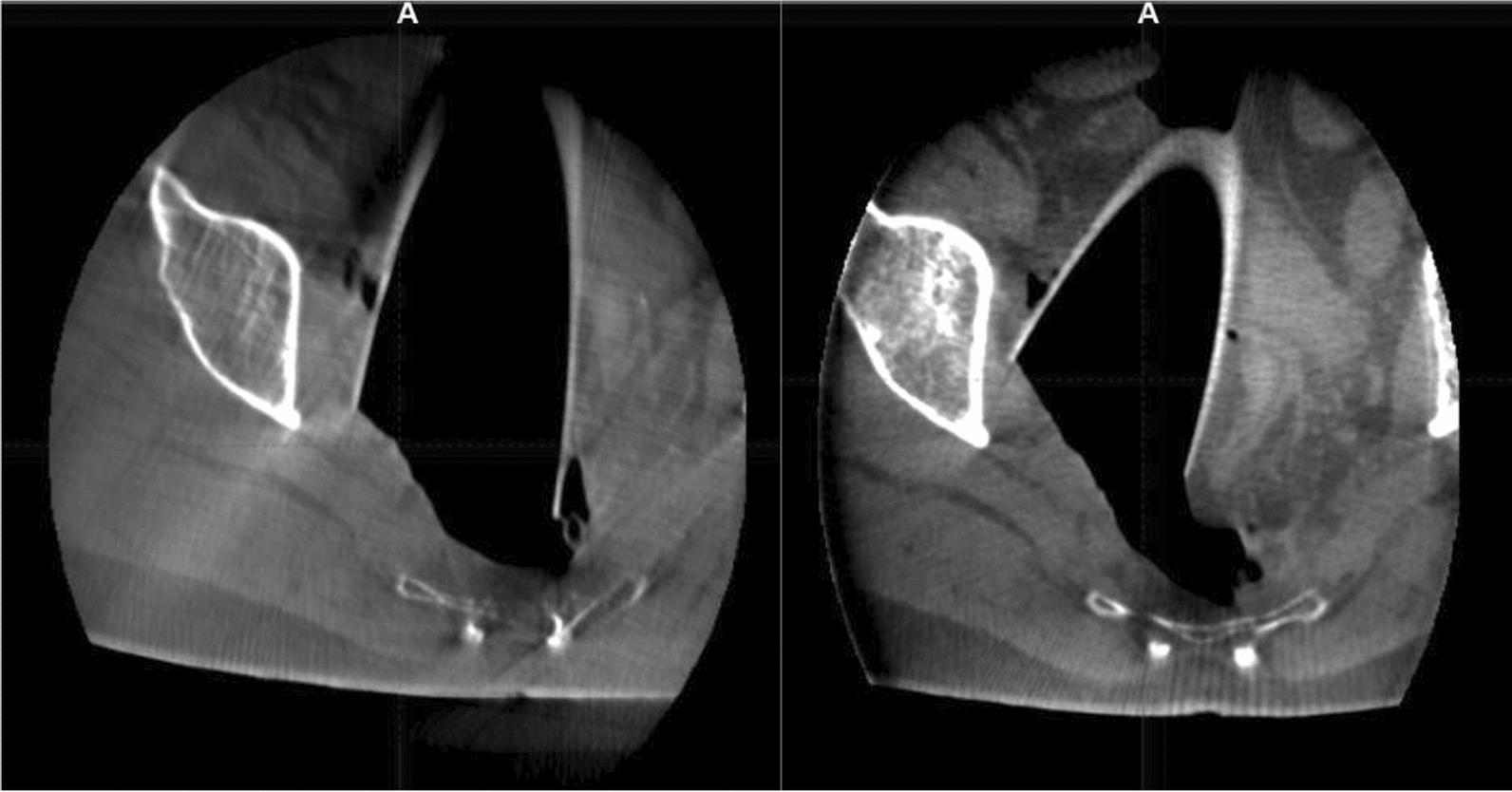
Cone-beam CT scans prior to and after correction of the applicator position

**Fig. 8:**
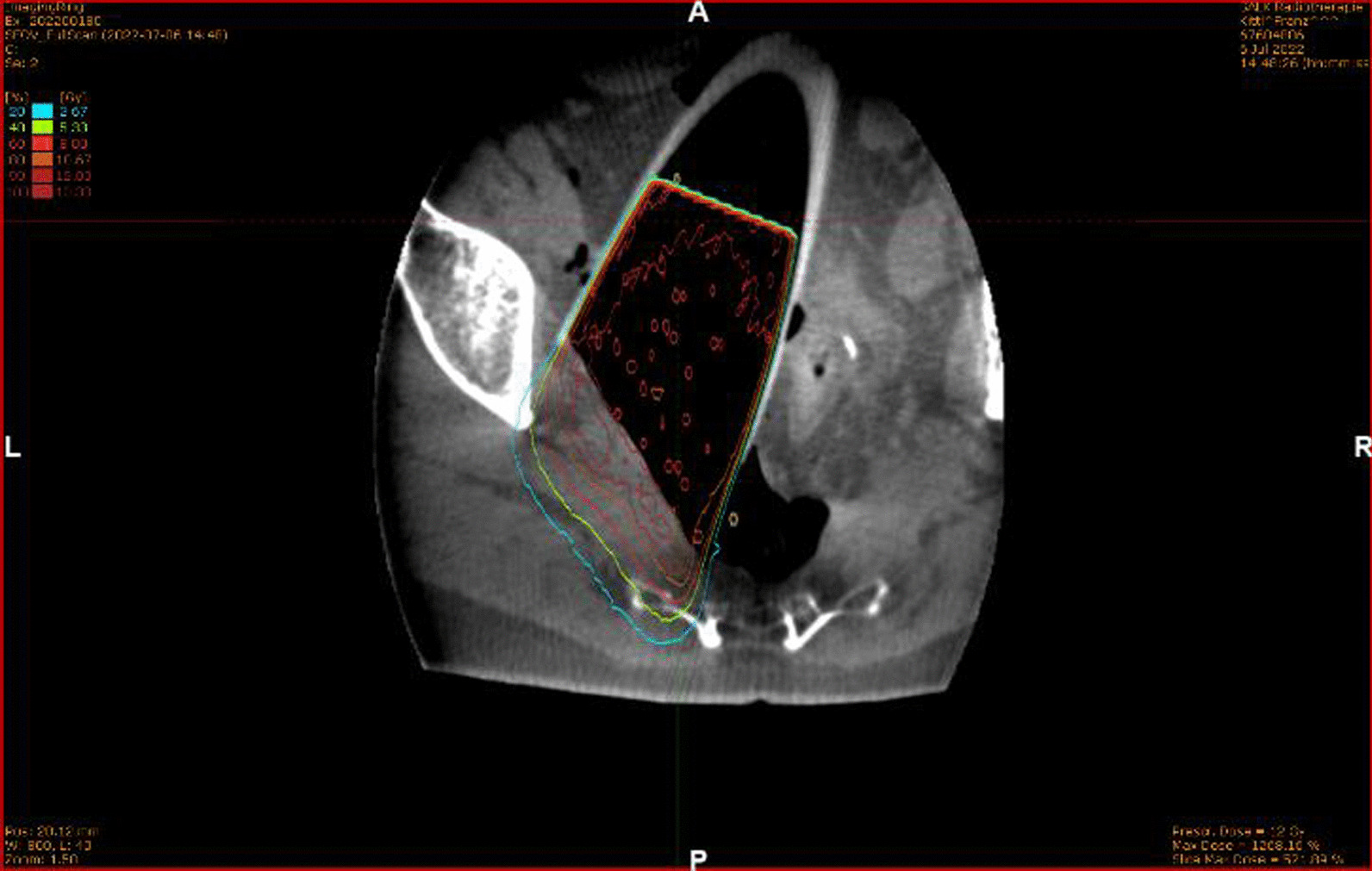
Dose distribution calculated on intraoperative cone-beam CT

**Fig. 9:**
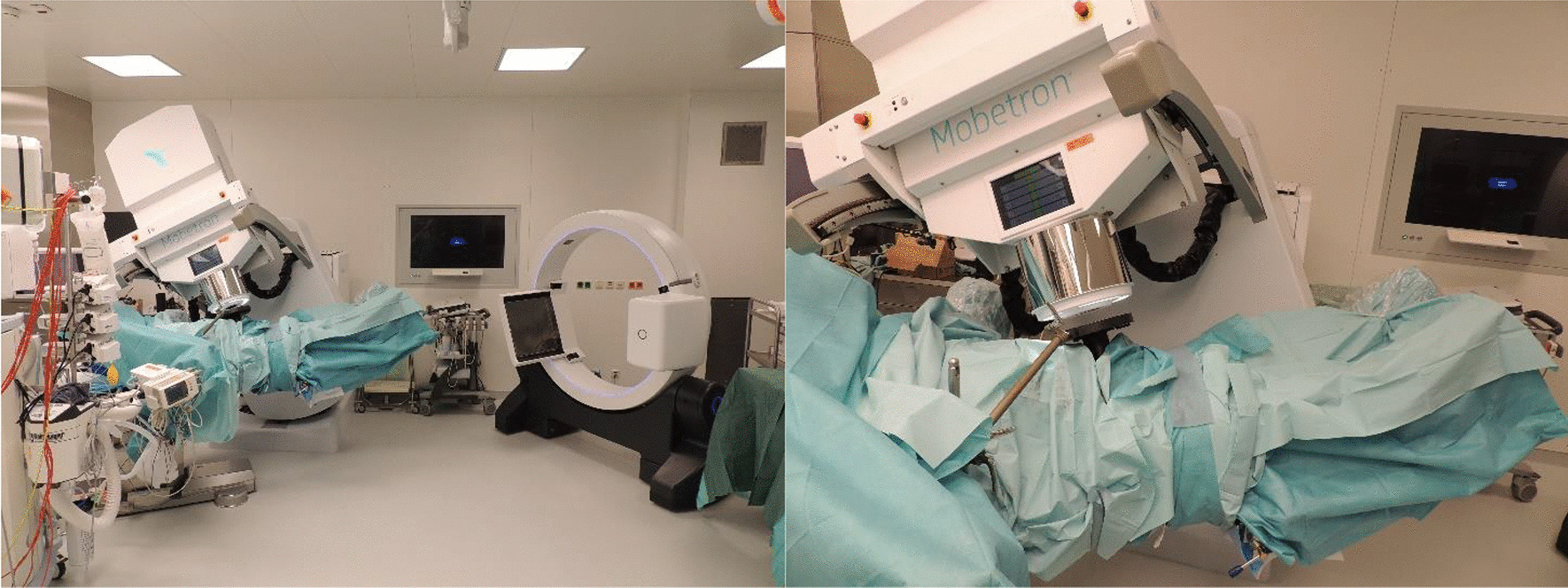
Patient in treatment position after soft-docking

Prior to surgery, parts of the table top of the surgical table were removed and replaced by a non-metal containing insert which is part of our carefully selected table setup (Maquet, Getinge) for intraoperative imaging purposes (Fig. 4). The patient was positioned in supine position as usual for abdominopelvic surgery using a median laparotomy with the pelvis located on the insert. Surgery was performed in our dedicated IOERT suite at the department of radiation oncology by a visceral surgeon specialized in the treatment of colorectal cancer. Gross total resection was achieved including partial resection of the directly adjacent right ureter, but margins to the pelvic side wall were very close according to the surgeons judgement. A circular shaped 30° beveled IOERT applicator (Polyoxymethylene, POM-C) of 6 cm diameter was placed to cover the tumor bed by the surgeon and the radiation oncologist together (Fig. 5). Bladder, rectal stump and both ends of the right ureter were securely placed outside the irradiation area. All dispensable metal-containing surgical equipment was removed and the patient was wrapped with sterile covers (Fig. 6). The mobile ImagingRing (medPhoton GmbH), which is a moveable cone-beam CT scanner with a large effective bore of 102 cm diameter capable of covering a field of view (FoV) of 49.1 × 49.1 × 25.4 cm^3^, was positioned around the patient above the applicator (Fig. 6). The large FoV allows the mapping of the tumor bed, partially the IOERT applicator and the surrounding anatomical structures. Two orthogonal X-ray images were taken prior to 3-D imaging to define an appropriate elliptically shaped scanning volume, which is subsequently captured in the course of CBCT acquisition using the dynamically moveable independent arms and four independently moveable collimator jaws to accurately image the region of interest (ROI) from all encountered viewing angles. The image acquisition system is further equipped with a time-of-flight laser for collision detection. As the system cannot distinguish whether the obstacle of a possible collision is rigid or flexible, all sterile covers were placed inside the cylinder defined by the effective gantry bore and a dry run was performed prior to the essential imaging to prevent a stop in rotation during imaging. CBCT images were acquired (Fig. 7) via remote control from outside the operation room. They showed a slightly incorrect applicator position based on comparison with the preoperative imaging, therefore the applicator was moved accordingly (Fig. 7). A second CBCT scan revealed a correct applicator position (Fig. 7) and was transferred to our treatment planning system (Radiance, GMV). Based on the surgeons assessment of a gross total resection and to prevent neuropathy in the directly adjacent sciatic nerve, the prescription dose was restricted to 12 Gy [1, 25, 26]. The mobile ImagingRing features an automated scaling (heuristic object and head scatter correction as well as beam hardening correction) of Hounsfield Units (HU), which was checked for the used imaging preset prior to clinical introduction by suitable standardized phantoms with inserts of differing densities (Lung: − 774HU ± 90HU, soft tissue: − 43 ± 58, bone: 712HU ± 221HU). This allows the application of one density conversion table independent of imaging preset, geometry, and patient anatomy, for standard clinical cases (in particular predominantly water equivalent tissue and low artefact disturbance). Dose calculation in the TPS (Radiance) uses a Monte Carlo algorithm and is based on a beam model consisting of phase-space files (PSF) created from water phantom measurements. The PSF was shortened 4 cm in front of the end of the tube by the manufacturer to take into account the volume of tissue and air within the tube (Fig. 8). In the current case, a dose distribution attempting to cover a tissue depth of 1 cm for the elliptic sectional plane under the tube with 12 Gy (corresponding to the 90% isodose) and the adequate monitor units using 9 MeV electrons was calculated (Fig. 8). During dose calculation, the patient was moved beneath the linear accelerator (Mobetron, IntraOP) including all anesthesia equipment (Fig. 9). After automated soft-docking, intraoperative irradiation was performed via remote control from outside the operation room, while the patient was monitored via video. After finishing the irradiation treatment, the patient was moved to the initial position and the applicator was removed from the patient. Surgery was finished by the visceral surgeons including a reconstruction of the partly resected ureter using the psoas-hitch technique by a urological surgeon.

### Follow-up

Postoperative complications included bacteremia with elevated inflammation blood parameters but without fever and repeated episodes of constipation. All were treated without interventions by pharmacological therapy only (Clavien-Dindo Grade 2). Moreover, the patient developed transient bladder incontinence, which resolved within 6 months from surgery. No signs of neuropathy have been observed so far. After a follow-up of 10 months, the patient shows no evidence of disease (NED) based on repeated restaging with pelvic MRI and PET-CT.

## Discussion

We present the first clinical application of an image-guided abdomino-pelvic IOERT with real time dose calculation in a patient suffering from recurrent rectal cancer. Various attempts have been made in the past by different research groups to overcome one of the main limitations of IOERT, namely the inability to perform dose calculations similar to the standards of EBRT. While all prior approaches using surface-scanning, orthogonal X-rays or ultrasound fell short with regard to dose calculation, (CB)CT-based approaches suffered either from unsuitable image quality due to artifacts or needed transportation of the patient outside the operating room. Herein, we describe a novel workflow for image guided IOERT using a mobile CBCT scanner to overcome the limitations of previously used techniques and approximations.

In contrast to the mentioned prior attempts, the described procedure allows a confirmation of the correct applicator position (or its adjustment if needed) and a visualization of the 3D dose distribution based on the individual intraoperative anatomy shown by the acquired CBCT images. This will further increase the precision of the dose guidance to the target as positioning of the applicator does not solely rely on the visual perception of the surgeon and/or the radiation oncologist anymore but can be verified by comparison of intra- and preoperative imaging. Possible influences of surface or tissue inhomogeneities as well as air gaps on target coverage or dose to organs at risk can be visualized and addressed by changing the treatment parameters if necessary. Moreover, a precise documentation of the intraoperatively applied dose distribution is now possible, allowing a more accurate summation of the intraoperative dose with the EBRT dose distribution within combination approaches.

**Fig. 10:**
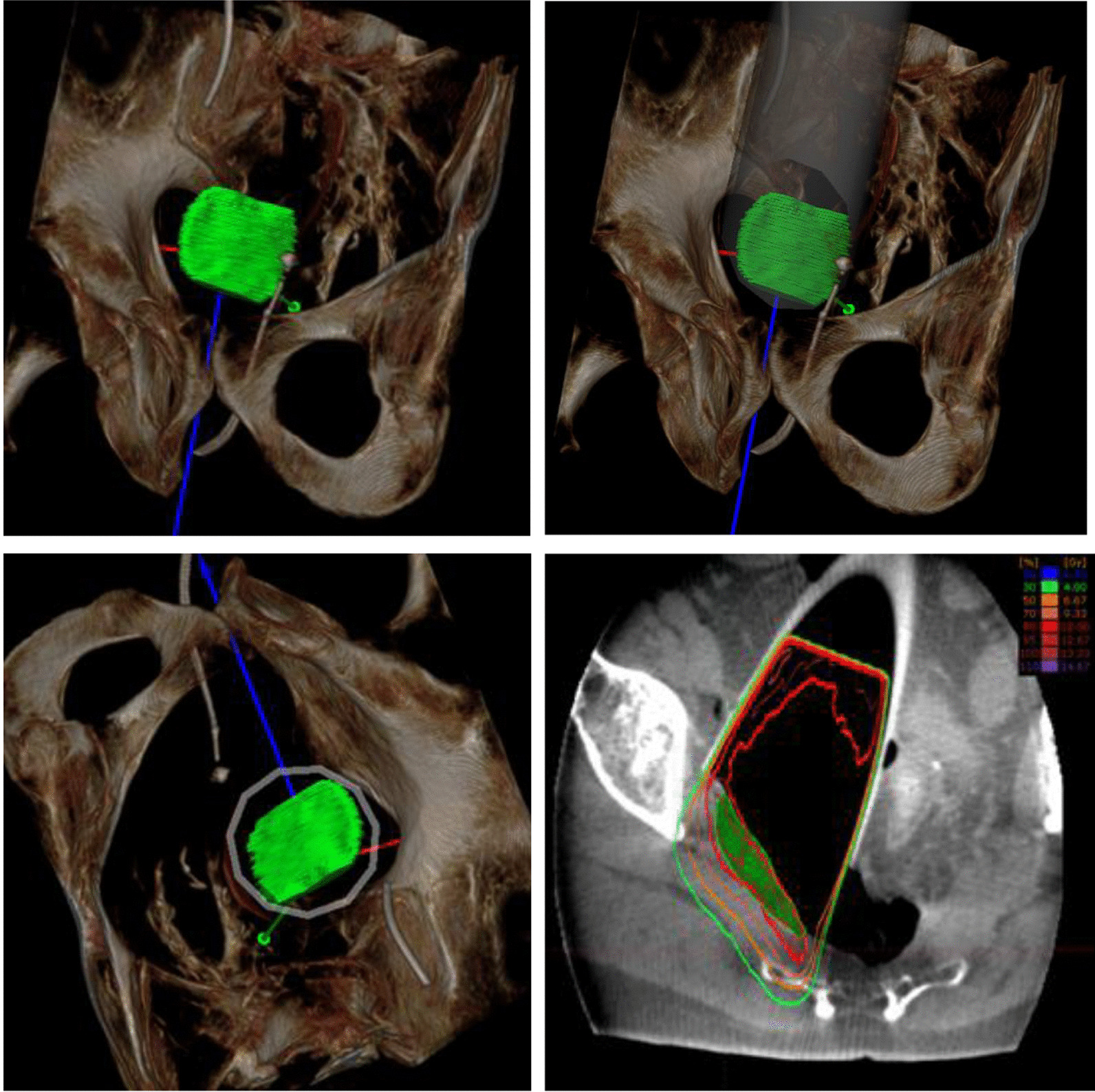
Postplanning with target volume delineation based on intraoperative imaging (upper left: 3D view of the target volume, upper right: 3D view with applicator, lower left: 3D beam eye view, upper right: axial slice with target volume and dose distribution as applied intraoperatively)

Due to time reasons and a current lack of possibility for fast and precise image fusion of intraoperative and preoperative imaging, intraoperative target volume delineation was not performed in this first case. However, we performed a 3D postplanning with delineation of the target volume based on the intraoperative images (see Fig. 10) after surgery. The adoption of the current work flow to routinely include intraoperative target volume delineation based on fused pre- and intraoperative images is current work in progress. This will further enhance treatment and dosimetric precision and allow treatment planning equal to EBRT standards.

Similar imaging approaches have been evaluated for intraoperative radiation therapy using HDR-brachytherapy or kV photons.

For example, Showalter et al. [27] reported a phase I trial evaluating intraoperative imaging in 28 patients receiving HDR-brachytherapy for breast cancer. After placement of a multilumen balloon catheter system, intraoperative images were acquired by a CT on rails and used for calculation of a customized brachytherapy plan. They reported a median IOERT time of 67 min (50–108) with a median planning time of 39 min and a median RT delivery time of 26 min. Corrections of the applicators after CT were needed in roughly 25% of the patients, mainly due to large air cavities and/or poor tissue conformity [28]. The prespecified planning goals were achieved in 79% of the patients.

Hassinger et al. [29] reported on 103 patients treated with the same procedure including the patients from the phase I trial and 75 patients from a subsequent phase II trial. In contrast to the phase I trial, transportation of patients from the operation room to a non-dedicated CT for intraoperative imaging as well as delayed application of the irradiation within 30 days from surgery was allowed in the phase II trial. The median total procedure time was 147 min including a median planning time of 48 min and a median IORT delivery time of 26 min. Similarly to the phase I trial, 26% of the patients needed an applicator adjustment. However, changes made to the initial dosimetry plan were necessary in 79% of the patients, mainly to reduce dose to skin or chest wall. The authors concluded that their technique seems to be superior to prior techniques through avoidance of applicator placement errors and the ability to customize radiation dosimetry to minimize dose to adjacent organs at risk.

Schneider et al. [30] reported the use of intraoperative CBCT images for the 50 kV Intrabeam System during kyphoplasty of a patient with a bone metastasis of a thoracic vertebra. For imaging purposes, only the needle applicator was inserted into the metastasis without the usually present connection to the small X-ray source. Intraoperative CBCT imaged were acquired in prone position and rigidly registered with preoperative CT scans in supine position. The applicator was contoured on the CBCT scan while all other structures were contoured on the preoperative CT scan. Dose calculations were performed with the Radiance treatment planning system on both imaging sets but showed major deviations up to 50% in the low and high dose region. The authors concluded that image guided intraoperative IORT is generally feasible, but that their current set-up is limited by CT artifacts if only the CBCT images are used. Therefore, they recommend image fusion of the intraoperative images with a preoperative CT scan to allow for an accurate dose calculation with the knowledge of the correct applicator position.

Although comparing treatment times and accuracy rates seems difficult within the presentation of one case, we assume that overall treatment time was less than the median time given in the report by Hassinger et al. [29] for intraoperative brachytherapy of breast cancer even with correction of the applicator. This may be caused by lower efforts for applicator placement and clearly lower treatment times with electrons compared to HDR-brachytherapy. Rates of applicator corrections cannot be fruitfully compared (single case, different body sites). Comparisons of dose distributions with virtual planning on preoperative CT scans as shown by Schneider et al. [30] was not done because we generally question the precision and validity of the virtual treatment planning approach and do not use it within our clinical workflow. However, the results of all of the mentioned studies including our own strengthen the rationale and need for intraoperative CT-based imaging to improve the quality of care for IORT procedures irrespective of the used technique.

Some limitations of our current work flow have to be addressed: Intraoperative CBCT images are suitable for confirmation of the applicator position and dose calculation but are (consistently with their use in EBRT) hardly usable for diagnostic purposes due to the current limitations in soft-tissue contrast visualization. Acquisition of the CBCT images requires an adequate position of the patient prior to surgery. For example, the ROI must not be placed above the column of the operation table. Even if properly positioned, one may need some adjustments of the patient position during surgery prior to imaging. For example, elevated legs (often used for pelvic surgery) or extended arm positions (usually used for axillary sentinel node resections during breast cancer surgery) are less suitable for imaging. Therefore, close multidisciplinary collaboration and discussion prior to and during surgery are mandatory. Moreover, CBCT image quality might be affected by metal artifacts either from surgical/IOERT equipment or by implants. Metal-containing IOERT applicators are not suitable for this approach and must be replaced by synthetic or plastic applicators. All dispensable metal-containing surgical equipment should be removed or placed as far away from the imaging region as possible. Metal-containing parts of the operation table (at least in the ROI) have to be replaced by non-metal inserts as described. The same is true for metal-containing parts of the fixation system. The heuristic scatter correction and HU mapping are mainly designed for standard situations [31]. Significant artifacts (for example due to relevant volumes of metal within the field of view) may result in deviations of the HUs. For an appropriate dose calculation, these have to corrected by manual HU assignment to particular volumes. In the reported case, no such corrections were necessary. Target volume delineation is currently only available based on intraoperative images and will be incorporated into our work flow soon. Delineation based on fused intraoperative and preoperative images is not yet available but represents work in progress.

## Conclusion

In summary, we report the first case of an image-guided IOERT with intraoperative real time dose calculation based on the individual intraoperative patient anatomy in the abdominopelvic region. This technique enables IOERT procedures adherent to known standards of EBRT or Brachytherapy and will further increase its treatment precision as well as its acceptance among radiation oncologists.

## Data Availability

The data presented in this study are available within the article.

## Abbreviations

3D: Three-dimensional
CBCT: Cone beam computed tomography
cm: Centimeter
CT: Computed tomography
EBRT: External beam radiation therapy
FDG-PET-CT: 18-F-fluorodeoxyglucose positron emission tomography computed tomography
FoV: Field of view
Gy: Gray
HU: Hounsfield units
IG-IOERT: Image-guided intraoperative electron radiation therapy
IMRT: Intensity-modulated radiation therapy
IORT: Intraoperative radiation therapy
IOERT: Intraoperative electron radiation therapy
m: Meter
MeV: Mega electron volt
mg: Milligramm
MRI: Magnetic resonance imaging
NED: No evidence of disease
POM-C: Polyoxymethylene
PSF: Phase-space files
ROI: Region of interest
TPS: Treatment planning system
VMAT: Volumetric intensity modulated arc therapy
